# *Pleurotus* spp.—an effective way in degradation mycotoxins? A comprehensive review

**DOI:** 10.1007/s12550-024-00572-z

**Published:** 2024-11-12

**Authors:** Agnieszka Zapaśnik, Marcin Bryła, Barbara Sokołowska, Agnieszka Waśkiewicz

**Affiliations:** 1 Department of Microbiology, Prof. Waclaw Dabrowski Institute of Agricultural and Food Biotechnology—State Research Institute, Rakowiecka 36, 02-532 Warsaw, Poland; 2 Department of Food Safety and Chemical Analysis, Prof. Waclaw Dabrowski Institute of Agricultural and Food Biotechnology—State Research Institute, Rakowiecka 36, 02-532 Warsaw, Poland; 3 Department of Chemistry, Poznan University of Life Sciences, Wojska Polskiego 75, 60-625 Poznan, Poland

**Keywords:** *Pleurotus*, Mycotoxins, Degradation, Ligninolytic enzymes, White rot fungi

## Abstract

Mycotoxins—secondary metabolites produced by filamentous fungal species—occur as a global problem in agriculture due to the reduction in crop quality and the negative effects on human and animal health. There is a need to develop environment-friendly methods of detoxification. In recent years, a number of biological methods for the removal/degradation of mycotoxins have been described. One of them—particularly interesting due to its high effectiveness—is mycoremediation, which involves the ability of *Pleurotus* spp. mushrooms to remove toxic contaminants from the environment and food. *Pleurotus* spp. biosynthesizes ligninolytic enzymes, such as laccase and manganese peroxidase that are the main factors of enzymatic degradation of various pollutants, including mycotoxins. The degradation process of mycotoxins (especially aflatoxins) with the participation of isolated enzymes reaches approximately 30–100%, depending on the culture conditions, substrate, and mediators used. In the food industry, their application may include, among others, the detoxification of animal feed from mycotoxins or fermentation products (e.g., juices and wines). While these applications are promising, they require further research to expand toxicological knowledge and optimize their use. This review presents current research on this new and very promising topic related to the use of edible *Pleurotus* spp. mushrooms in the process of biological degradation of toxic fungal metabolites.

## Introduction

Mycotoxins—toxic secondary metabolites of filamentous fungi—have a diverse chemical structure due to the variety of heteroatoms, including functional groups (Lopes et al. 2023). Among this group, there are compounds that have a number of disease effects on living organisms—mutagenic (AFB_1_, FBs, OTA), teratogenic (AFB_1_, OTA), nephrotoxic (OTA), hepatotoxic (AFB_1_, OTA), and strong estrogenic (ZEN) (Elkenany and Awad 2021; Awuchi et al. 2022; Da Silva et al. 2022). In addition, several of them have carcinogenic properties—according to the classification of the International Agency for Research on Cancer (IARC 2012), they include aflatoxins (group 1A—carcinogenic to humans) and the sum of FB1 and FB2, as well as OTA (probably carcinogenic to humans—group 2B) (Ostry et al. 2017). Out of concern for the high quality of food and consumer health, effective food purification methods to remove mycotoxins are being sought that will not adversely affect the efficiency of production processes.

The chemical and physical methods of detoxification of food and feed are commonly used in food industry over decades, but their influence on various nutrition and sensory parameters is unfavorable and moreover may contribute to the reduction of the quality of final products. In recent years, attention has turned to mycoremediation, which is a technique that uses the ability of fungi to remove toxic contaminants by degradation from the environment and food in an environmentally and economically sustainable manner (Kapahi and Sachdeva 2017). This technique may be a very promising method for reducing food contamination with harmful compounds, including mycotoxins (Chang and Chang 2016; Boamponsem et al. 2013; Vaseem et al. 2020; Brana et al. 2017; Ergun and Urek 2017). There is a specific group of fungi—White Rot Fungi (WRF), which include organisms characterized by their ability to produce ligninolytic enzymes. Taking into account the wide use of *Pleurotus* spp. in agricultural waste management (including the use of low-quality cereals withdrawn from commercial circulation due to failure to meet standards regarding the permissible content of mycotoxins), we focus on their potential for the biological decomposition of mycotoxins.

*Pleurotus* spp. are edible mushrooms cultivated on various lignocellulosic substrates and classified into the Basidomycetes division and Agaricomycetes class. Over the last two decades, they have gained great popularity both among consumers and in the scientific area due to their health-promoting potential and extensive enzymatic system (Tellez-Tellez and Diaz-Godinez 2019; Torres-Martinez et al. 2022). *Pleurotus* spp. synthesize enzymes derived from several groups used in the biotechnology industry, i.e., amylases, catalases, cellulases, lipases, oxidoreductases, pectinases, and proteases (Tellez-Tellez and Diaz-Godinez 2019).

The use of cereals contaminated with mycotoxins as a substrate for the cultivation of *Pleurotus* spp. may be a very promising biological method of removing/degrading these xenobiotics. Therefore, the research presented so far on this topic will be the first comprehensive presentation of available knowledge about the effectiveness of these mushrooms.

## Fungal diseases of cereal crops

Cereals are basic raw materials for food and feed production worldwide, the cultivation of which is associated with a high risk of the development of fungal diseases and the production of mycotoxins that contribute to a reduction in the quantity and quality of the yield and consequently to large economic losses (Fleurad-Lessard 2015). It is estimated that yield losses oscillate between 15 and 20%, and in extreme cases, they reach up to 50% (Eskola et al. 2019; Różewicz et al. 2021).

The division of filamentous fungi is determined by the conditions that favor their growth. Two groups are distinguished, i.e., phytopathogenic and saprophytic fungi. The first group includes *Fusarium* spp. that infect plants during the growing season and biosynthesize a number of mycotoxins (fumonisins (FBs), deoxynivalenol (DON) with derivatives, and zearalenone (ZEN)) that deteriorate the health and commercial quality of cereals (Ji et al. 2019). The most important factors influencing the development of *Fusarium* spp. are limited crop rotation and climate change, as well as excessive use of nitrogen fertilizers and monoculture cultivation (Różewicz et al. 2021). Among cereal diseases, *Fusarium* head blight dominates, as well as root rot and seedling wilt (Shude et al. 2020; Różewicz et al. 2021).

The second group is made up of saprophytic fungi (*Aspergillus* and *Penicillium* genera), which mainly develop under storage conditions (Alshannaq and Yu 2017) and produce mycotoxins including aflatoxins (AFB_1_, AFB_2_, AFG_1_, AFG_2_, AM1), patulin (PAT), ochratoxin A (OTA), citrinin, and many others (Egbuta et al. 2017). The proliferation of saprophytic fungi is related to storage conditions—the high moisture content of grain, as well as the high temperature and long storage period, stimulate the development of these fungi (Fleurad-Lessard 2015). The effects of the development of *Aspergillus* and *Penicillium* genera during grain storage are losses in the dry mass of the grain, grain cracking, a reduction in nutritional value, and the presence of toxic metabolites (Fleurad-Lessard 2015).

Both of these groups contribute to mycotoxin productions in different kinds of food matrices, which in turn leads to negative health’s effects among consumers as well as great economic losses.

## Impact of mycotoxin contamination and detoxification strategies

Contamination of food and feed with mycotoxins is a worldwide problem with adverse effects on human and animal health and large economic losses. Cereal infections can occur during vegetation, harvest, transport, processing, and storage (Haidukowski et al. 2019; Martin et al. 2022). In recent years, approximately 10,000 species of potentially toxigenic filamentous fungi and more than 500 secondary metabolites with toxic properties that threaten human health have been identified (Haque et al. 2020). An estimated 25–50% of the world’s food products are contaminated with mycotoxins at concentrations that contribute to reduced commercial and health-related food quality (Haque et al. 2020). Mycotoxins can occur in a wide range of plants, including corn and other grains, vegetables, fruits, nuts, and their products. They can also enter the human gastrointestinal tract indirectly through consumption of milk or meat from livestock fed contaminated feed (Tola and Kebede 2016; Alshannaq and Yu 2017; Garzia-Das et al. 2020; Abdolmaleki et al. 2021).

Table 1 shows the characteristics of the most common mycotoxins, as well as information on their producers, source, impact on human health, and the TDI (tolerable daily intake).

**Table 1:** Main mycotoxins, their producers and sources, effects on human health, and TDI (tolerable daily intake) values

Mycotoxins	Food matrix	Fungi species	Health damage	TDI	References
Fumonisins	Maize, grapes	*Fusarium proliferatum*,* Fusarium verticillioides*	Teratogenic, carcinogenic, cytotoxic	2 µg/kg bw	Jabir et al. (2019); Taheur et al. (2019); Pitt et al. (2012)
Aflatoxins	Milk, maize, rice, peanuts, barley, cocoa beans, nuts	*Aspergillus flavus*, *Aspergillus parasiticus*,* Aspergillus nominus*	Carcinogenic, genotoxic, hepatotoxic, DNA damage, teratogenic	No estimated level. Exposure should be as low as possible	Karlovsky et al. (2016); Taheur et al. (2019); Pitt et al. (2012)
Ochratoxin A	Maize, barley, wheat, legumes, nuts, coffee, cocoa, spices, seeds, dried fruits	*Aspergillus carbonarius*, *Aspergillus ochraceus*, *Penicillium verrucosum*	Nephrotoxic, carcinogenic, immunotoxic, hepatotoxic	14 ng/kg bw	Jabir et al. (2019); Taheur et al. (2019); Pitt et al. (2012)
Zearalenone	Cereal grain	*Fusarium roseum*, *Fusarium graminearum*, *Fusarium culmorum*,* Fusarium oxysporum*	Endocrine disorders	0.25 µg/kg bw	Karlovsky et al. (2016); Pitt et al. (2012)
Deoxynivalenol	Barley, maize, wheat, oats, rice	*Fusarium culmorum*, *Fusarium graminearum*	Vomiting, diarrhea, and risk of heart, liver, and immune system disorders due to high doses	1 µg/kg bw	Karlovsky et al. (2016); Pitt et al. (2012)

Based on the 2020 RASFF report, there were 400 notifications about the presence of mycotoxins in food, with AFB_1_ being the most frequently detected toxin. Most notifications about the presence of mycotoxins were recorded for samples of nuts, seeds, herbs, spices, fruits, vegetables, and bakery products (Annual Report RASFF n.d.). Due to the high toxicity of these metabolites, food producers from the European Union countries are obliged to comply with the current legal limits regarding maximum permissible levels of these compounds in food, contained in the European Commission Regulation (EC 2023/915 n.d.). Control of the presence of these metabolites in food is carried out in two ways, i.e., prevention of the growth of toxigenic filamentous fungi and detoxification of contaminated raw materials.

Taking into account the risk of mycotoxins in the food chain, detoxification of contaminated food is an extremely important and topical issue. Heat treatment can be highlighted among the physical methods used to reduce the level of mycotoxin inactivation. However, fungal toxins exhibit thermal stability, which affects the low efficiency of this process (Colovic et al. 2019). Some mycotoxins (AFs) show stability even at very high temperatures (i.e., above 200 °C), and their degree of degradation during thermal treatments is low. Others show moderate stability, undergoing partial degradation under temperatures ranging from 120 to 180 °C (Kabak 2009; OTA, ZEN, DON) (Milani and Maleki 2014). Moreover, it should be remembered that thermal decomposition products may be more toxic than the parent forms. Physical methods do not guarantee satisfactory efficiency and further affect the deterioration of food quality due to heat treatment at high temperatures. In turn, the use of chemical methods to detoxify mycotoxin-contaminated food has positive effects, but it also has limitations due to the potentially negative impact on the safety and quality of food products. Codex Alimentarius defines a list of chemicals that can be used during technological processes to reduce mycotoxins (Codex Alimentarius 2007; Karlovsky et al. 2016). Chemical detoxification methods include the use of ammonia, acids (phosphoric acid, acetic acid, propionic acid, formic acid, sorbic acid), bases (sodium hydroxide, calcium hydroxide), and oxidizing (hydrogen peroxide, ozone) and reducing (sodium bisulfate) chemicals (Varga et al. 2010).

## Biological methods for mycotoxin degradation

Numerous literature data indicate that biological methods of mycotoxin detoxification that rely on the detoxifying properties of selected microorganisms are a promising alternative to classical physical and chemical methods. Biological methods are associated with a lower risk of negative effects on the health and technological quality of food and are characterized by high efficiency against various mycotoxins. An acceptable/effective mycotoxin detoxification method should be characterized by a wide range of action against various types of fungal toxins, high effectiveness in degrading or inactivating toxins without the risk of forming toxic by-products, and no negative impact on the nutritional value, technological, and sensory properties of food and feed (Colovic et al. 2019).

Many authors have also analyzed the possibility of using microorganisms to reduce mycotoxin level, focusing primarily on lactic acid bacteria or yeast (Król et al. 2018; Fu et al. 2021), as well as the use of natural plant extracts rich in bioactive compounds (Uwineza et al. 2022). The action of microorganisms is based on inhibiting the growth of pathogenic fungi by competing for nutrients and producing metabolites with antimicrobial properties, which can include organic acids and antibiotics (Ruggirello et al. 2019). Many studies have proven that lactic acid bacteria and yeast exhibit such effects (Lipinska et al. 2016; Ruggirello et al. 2019), as well as bacteria of the *Bacillus* or *Enterobacter* genus (Wiraswati et al. 2019). In addition, particular strains exhibit detoxifying activity through the biotransformation of toxins into a product of less toxicity or bioadhesion to the cell wall of the microorganism in question. Many authors have demonstrated the effectiveness of lactic acid bacteria in degrading mycotoxins in vitro, using their ability to absorb toxins into the interior of bacterial cells or bind to the cell wall (Zhou et al. 2017; Rogowska et al. 2019; Złoch et al. 2020). There are also studies on the usefulness of other microorganisms in neutralizing fungal toxins, including the bacteria *Bacillus licheniformis* (Raksha et al. 2017; Wang et al. 2020) and *Bacillus amyloliquefaciens* (Lee et al. 2017), as well as the yeasts *Saccharomyces cerevisiae* (Armando et al. 2012) and *Candida intermedia* (Farbo et al. 2016). Unfortunately, most of these reports in the literature focus on in vitro tests, which are sometimes not valid for in vivo conditions. The effectiveness of biological methods using microorganisms may be much lower due to the specific parameters of the food matrix (pH, chemical composition of the food, subsequent technological treatments).

Among biological methods, the use of ligninolytic enzymes, whose detoxifying effects have been described against mycotoxins, should also be noted. According to a study by Qin et al. (2021), some mycotoxins are structurally similar to lignin monomers or related compounds, suggesting the usefulness of using ligninolytic enzymes to degrade particular fungal toxins. Moreover, due to their low specificity of action, ligninolytic enzymes can be effective for detoxifying a wide range of contaminants, including mycotoxins (Rodríguez-Couto 2017). Laccase and manganese peroxidase are produced by various species of wood-decaying fungi. WRF are organisms capable of mineralizing the refractory heteropolymer lignin present in wood. This ability is related to the ligninolytic enzyme complex (lignin peroxidase, manganese peroxidase, and laccase), which is produced as a result of secondary metabolism (Rodríguez-Couto 2017). WRF organisms have two types of extracellular systems—the first is a hydrolytic system that produces hydrolases responsible for the degradation of polysaccharides, and the second is a ligninolytic system—important for mycotoxin detoxification—that degrades lignin and leads to the opening of phenyl rings (Ergun and Urek 2017). The group of WRF organisms includes fungi of the genera *Trametes*, *Phlebia*, *Coriolopsis*, and *Pleurotus*. The *Pleurotus* genus represents a promising group of commercially produced organisms capable of synthesizing ligninolytic enzymes with proven mycotoxin detoxification potential.

## Characterization of *Pleurotus* spp.

The morphological structure of *Pleurotus* spp. consists of mycelium (vegetative phase) and fruiting bodies (reproductive phase) (Sharma et al. 2021). *Pleurotus* spp. are a rich source of bioactive substances (polysaccharides, lipopolysaccharides, peptides, glycoproteins, triterpenoids, nucleosides, and lectins) (Tolera and Abera 2017) and widely available and consumed, especially in Asian and European countries, due to their abundance in nutrients and low production costs (Mahari et al. 2020).

Their growth usually uses organic matrices high in lignin, cellulose, and other polysaccharides for growth, which they decompose enzymatically. They are characterized by rapid growth and the ability to develop on a variety of substrates, including agrowastes, i.e., wheat, rice, and soybean straw; pine needles; banana and hazelnut leaves; sunflower and corn stalks; sugarcane bagasse; spent tea; or bamboo leaves (Hossain 2017). On an industrial scale, cultivation of *Pleurotus* is easy and inexpensive due to the inexpensive cost of the raw material for culture (Raman et al. 2021; Fufa et al. 2021).

The cultivation of *Pleurotus* spp. is based primarily on their adaptation to the composition of the substrate, mycelial proliferation, and the release of fruiting bodies. Commercial cultivation of mushrooms involves several steps, which must be carried out with special care to avoid microbial contamination of the material or damage to the mycelium. The required conditions usually assume an air humidity of 80–90% and a temperature oscillating between 20 and 30 °C during the initial phase of mycelial hypertrophy, which should be lowered to 17–20 °C during the phase of fruiting body formation (Fufa et al. 2021; Houette et al. 2022). This is confirmed by a study by Hoa and Wang (2015), which tested the effects of different temperature variants (16–36 °C) on mycelium growth. They found that temperatures of 16 and 36 °C had a negative effect on mycelium growth, while 28 °C was the most favorable. Similar results were obtained by Pereima and Ivanova (2017), in a study where a temperature of 28 °C was optimal for mycelium growth. In addition to ensuring adequate temperature, humidity, and access to air and light, the composition of the substrate (pH, carbon/nitrogen ratio, minerals, water activity, moisture), as well as the quantity and quality of the inoculum, are also important (Bellettini et al. 2019). *Pleurotus* spp. are saprophytes that obtain nutrients (carbon, vitamins, and minerals) from the substrate via mycelium; hence, their choice is important (Sharma et al. 2013; Hoa and Wang 2015; Rambey et al. 2020). By providing a high-quality substrate previously stored under appropriate conditions, the possibility of microbial contamination is reduced (Bellettini et al. 2019). *Pleurotus* spp. mycelium overgrows the substrate more quickly relative to green fungi, i.e., *Aspergillus* and *Trichoderma*, for which such substrate is nutrient deficient (Meija and Alberto 2013). Some authors investigated the effect of the presence of different carbon sources (glucose, dextrose, fructose, maltose, sucrose) on the growth yield of *Pleurotus* spp. (Hoa and Wang 2015; Pereima and Ivanova 2017). Their observations suggest that sucrose at a concentration of 1–5% in the medium can stimulate mycelium growth (Hoa and Wang 2015; Pereima & Ivanova 2017). A study by Hoa and Wang (2015) found brown rice, yellow corn, and wheat to be the most favorable grains for mycelium growth. Rambey et al. (2020) confirmed that a mixture of rice straw and sawdust provides a good substrate for *P. ostreatus* growth. In turn, Sharma et al. (2013) also noted rice straw as a very good substrate for *Pleurotus* spp. mycelium growth, suggesting that mixtures of rice straw and wheat straw or sugarcane can be a good alternative to traditional substrates (sawdust). Substrate moisture content is a very important factor affecting mycelium growth and quality. Too high moisture content limits the porosity of the substrate and thus the availability of oxygen for the developing mycelium. This results in surface proliferation of the mycelium and allows competitive microflora to develop (Bellettini et al. 2019). According to Ryu et al. (2015), substrate moisture above 70% is conducive to mold growth. Too low humidity can inhibit mycelium growth or lead to its decay. Ensuring adequate pH of the substrate can stimulate mycelium growth and fruiting body release. According to Kalaw et al. (2022), the optimal pH conditions for the development of mycelium and fruiting bodies should oscillate between 5.0 and 8.0. Another study confirmed that the wide range of pH oscillating between 5.0 and 8.0 is most favorable for the development of *P. ostreatus* (Aubrey et al. 2022).

According to the available literature data, a few species of *Pleurotus* fungi seem specially promising; among others, *P. eryngii*, *P. pulmonarius*, and *P. ostreatus* are mentioned by many authors as capable of producing ligninolytic enzymes used in in vitro studies on the degradation of various mycotoxins (Jackson et al. 2017; Loi et al. 2028, Song et al. 2021).

## Role of ligninolytic enzymes produced by *Pleurotus* spp.

Laccase and manganese peroxidase—ligninolytic enzymes produced by *Pleurotus* spp.—are the most noteworthy from the point of view of degradation of mycotoxins—compounds biosynthesized by toxigenic species of filamentous fungi (Silva and Venancio 2020).

Laccases are glycoproteins classified as multicopper oxidoreductases that find use as catalysts for the oxidation of naturally derived compounds and xenobiotics, reducing an oxygen molecule to water (Díaz-Godínez et al. 2013; Loi et al. 2016). They are widely used in the biotechnology industry, including paper bleaching, decolorization, or environmental cleanup of pollutants. Fungi typically secrete several laccase isoenzymes. *Pleurotus* spp. encodes several genes responsible for laccase production, and the number of these genes depends on the species. In *P. ostreatus*, 12 such genes have been identified thus far, and among them, 6 have been isolated and characterized (Zhu et al. 2016; Jiao et al. 2018). These isoenzymes often exhibit different catalytic properties and regulatory mechanisms, as well as localization. Extracellular laccase is synthesized in low amounts under natural conditions, and its synthesis and secretion depend on culture conditions, nutrients, and developmental stage (Afreen et al. 2018). Laccase activity can be increased by physical (temperature, pH) and chemical (addition of aromatic or phenolic compounds related to lignin or metals) factors (Munoz et al. 1997; DeSouza et al. 2003; Yang et al. 2013; Díaz-Godínez et al. 2013; Araujo et al. 2021). Díaz-Godínez et al. (2013) tested the effect of culture substrate pH conditions on the synthesis and expression of laccase genes during the growth *of P. ostreatus* by submerged fermentation. According to the results, the initial pH of the culture substrate has a very significant effect on both the synthesis and expression of the laccase isoenzyme profile. The results indicate that an acidic pH (4.50) is more conducive to laccase biosynthesis than an alkaline pH (8.50). Similar results were obtained in a study by Munoz et al. (1997), where the optimum pH for laccase was 4.50. In recent years, attention has also been devoted to studying the effect of light on the growth of *Pleurotus* spp. in the context of laccase biosynthesis. Araujo et al. (2021) observed that green light can stimulate and inhibit laccase activity, depending on the species. The activity of this enzyme in *P. ostreatus*, *P. citrinopileatus*, and *P. pulmonarius* species significantly increased under green light illumination. For the other species tested, green light decreased laccase activity. The effect of aromatic or phenolic compounds on increasing laccase activity was confirmed in a study by DeSouza et al. (2003). Among the phenols tested, ferulic acid and vanillin induced laccase activity synthesized by *P. pulmonarius* to the greatest extent. Similar conclusions were reached by Yang et al. (2013), who confirmed the beneficial effect of compounds such as ferulic acid and veratric acid, vanillin, or xylidine on the production of laccase by *Pleurotus* spp. (Yang et al. 2013). Some metals also have a stimulatory effect on enzyme synthesis, and among these, most literature reports concern copper, significantly inducing laccase activity in almost all mushrooms (Zhu et al. 2016).

Manganese peroxidase is an oxidoreductase, and its action is based on hydrogen peroxide-dependent catalysis of lignin polymers and organic compounds, including environmental pollutants (Salame et al. 2013; Xu et al. 2017). In nature, peroxidase catalyzes plant lignin depolymerase and is used in agriculture to degrade cellulose, hemicellulose, and lignin (Xu et al. 2017). According to Fernández-Fueyo et al. (2014a), *P. ostreatus* has nine genes encoding lignin peroxidases—six manganese peroxidases and three versatile peroxidases. Similar to laccase, manganese peroxidase shows stability under acidic conditions (pH ≈ 5), but its activity can be inhibited when the pH exceeds a value below 4.0 or the enzyme can be completely inactivated at a pH of 3.0 (Fernández-Fueyo et al. 2014a). In an alkaline environment (pH ≥ 8.0), a gradual decrease in enzyme activity is also observed (Fernández-Fueyo et al. 2014a). Modifications in pH conditions have a greater effect on peroxidase expression and activity relative to temperature modifications. Extreme temperatures below 10 °C and above 35 °C also lead to inhibition of peroxidase expression and activity because these conditions do not promote mycelial survival (Fernández-Fueyo et al. 2014b). Some authors suggest that manganese peroxidase activity is closely related to the developmental stage and physiological state of the mushroom culture as well as depends on the species (Elisashvilie et al. 2003). In the case of another fungus belonging to the WRT group (*Phanerochaete chrysosporium*), the optimal temperature and pH conditions to promote synthesis and stimulate manganese peroxidase activity were 37 °C and pH 4.5, respectively (Urek and Pazarlioglu 2007). In a study by Elisashvilie et al. (2003), laccase and manganese peroxidase activities were checked during the growth of *P. ostreatus*. Laccase showed the highest activity during the stages of colonization and fruiting body formation until harvesting, after which activity decreased but increased again with the next cast of fruiting bodies. In contrast to laccase, manganese peroxidase activity was relatively high during the first stage of mycelial colonization and gradually declined during subsequent stages of fruiting body formation.

Due to the fact that most of the available literature data are focused on the laccase enzyme, there is an assumption that laccase demonstrates the higher activity in reduction of mycotoxins compared to manganese peroxidase, which is more describe in the next paragraph.

## Potential of *Pleurotus* spp. to degrade mycotoxins

Research on mycotoxin degradation using *Pleurotus* spp. has been described by only a few authors so far. It is being conducted in two directions and concerns the analysis of the effectiveness of ligninolytic enzymes (laccase and manganese peroxidase) isolated from either the fungi or the postculture substrate, as well as the ability of mycelium or fruiting bodies to absorb toxins from the contaminated substrate. Current data indicate that both directions seem promising for developing an effective method for biological neutralization/degradation of mycotoxins.

An in vitro study by Brana et al. (2017) demonstrated the potential of *P. eryngii* fungi to detoxify AFB_1_ under laboratory conditions. The degradation rate of the mycotoxin was checked using malt extract broth (MEB) liquid medium and malt extract agar (MEA) solid medium. In the first model (liquid medium), AFB_1_ degradation reached 100% after 30 days of incubation, while degradation on solid medium (MEA agar) reached a slightly lower level, oscillating between 71 and 94%. Available literature data indicate that among the enzymes produced by fungi of the *Pleurotus* genus, manganese peroxidase and laccase are the most important in terms of neutralizing mycotoxins. Several authors have focused on analyzing the activity of laccase isolated from *Pleurotus* fungi (Alberts et al. 2009; Song et al. 2021). A study by Loi et al. (2016) showed a significant effect of the laccase enzyme synthesized by *P. pulmonarius* fungi on the degradation of AFB_1_ and aflatoxin M1 (AFM1). AFM1 is a product of the catabolic transformation of AFB_1_, found in milk and dairy products and characterized by slightly lower toxicity compared to the original form. The authors proved the effectiveness of laccase in degrading both forms of aflatoxin but noted that the degradation process is enhanced in the presence of three redox mediators (three phenolic compounds: (SA) syringaldehyde, (AS) acetosyringone, and (ABTS) 2,2-azino-bis 3-ethylbenzothiazoline-6-sulfonic acid). Currently, most studies focus on AFB1 detoxification due to its high toxicity and confirmed detoxification efficiency under the action of ligninolytic enzymes. However, there is a lack of more detailed research that also focuses on other mycotoxins that contaminate cereals, feed, and food. A study by Loi et al. (2018) evaluated the detoxification capabilities of laccase Ery4 from *P. eryngii* species in the presence of several redox mediators against various mycotoxins (ZEN, DON, FB1, OTA, T-2). The highest degree of FB1 degradation (74%) was achieved by enhancing the laccase action with the addition of mediators ((TEMPO) 2,2,6,6-tetramethylpiperidin-1-yl, (PhR) phenol red). In the case of OTA, no significant degradation was achieved using either the enzyme alone or the enzyme-mediator complex; natural phenolic compounds enhanced the action of the enzyme, but the degree of degradation did not exceed 30%. In turn, ZEN was completely removed from the test material using the three most effective mediators in combination with laccase (TEMPO, (SA) syringaldehyde, (ABTS) 2,2-azino-bis 3-ethylbenzothiazoline-6-sulfonic acid), while DON proved to be the most difficult toxin to remove both with the enzyme alone and with the mediators. This research is important not only because it compares the effect of the addition of mediators on the efficacy of laccase in the context of mycotoxin degradation but also because it is one of the few papers where the enzyme detoxification capabilities against various mycotoxins are presented. In the study conducted by Song et al. (2021), the effectiveness of laccase from *P. pulmonarius* species in the context of AFB_1_ and ZEN degradation was analyzed. They found that laccase in the presence of the mediator ABTS and an alkaline pH was able to completely degrade both mycotoxins.

The effect of manganese peroxidase on selected mycotoxins was also tested, and it was shown that after a 24-h incubation in the presence of the enzyme, AFB_1_ was degraded by 67%, and after extending the process to 48 h, detoxification reached 90%, where in both cases, the enzyme concentration was 1.5 U/mL (Yehia 2014). It was also demonstrated that enzyme activity can be enhanced in the presence of calcium, copper, and manganese cations. Wang et al. (2019) investigated the activity of manganese peroxidase isolated from WRF fungi (*Phanerochaete chrysosporium*, *Irpex lacteus*, *Ceriporiopsis subvermispora*, *Nematoloma frowardii*) in the context of removing selected mycotoxins (AFB_1_, DON, ZEN, FB1). It was confirmed that the enzyme is capable of degrading the above toxins only in the presence of dicarboxylic acid malonate.

The research conducted by Brana et al. (2020) compared the effectiveness of the two enzymes and the effect of incubation conditions on the degradation of fungal toxins, assessing the suitability of the spent mushroom substrate for AFB_1_ degradation. SMS (spent mushroom substrate) is rich in ligninolytic enzymes synthesized by mushrooms during the culture period. The authors confirmed the positive effect of these enzymes on AFB_1_ reduction in vitro. More than 90% of the toxin was degraded after 7 days of incubation, and analysis of the efficacy of both enzymes showed that laccase had a higher activity than manganese peroxidase. In the same experiment, the effect of temperature and pH on the level of degradation was evaluated. It was found that temperature oscillating between 25 and 37 °C and pH ≥ 8 were most conducive to the degradation process. On the other hand, Nobre et al. (2022) investigated the degradation potential of *P. ostreatus* (powder) to detoxify OTA and ZEN during the gastrointestinal digestion process. The study considered the effect of pH conditions on the efficiency of the detoxification process and attempted to identify the degradation mechanisms of individual toxins. The culture was carried out on commercially available sorghum medium, which was then inoculated with sorghum grains overgrown with *P. ostreatus* mycelium. The culture process took 40 days at 28 °C without light. The harvested fruiting bodies were dried and then ground to begin the in vitro stage of testing. Experiments were conducted at pH values of 3.0, 5.0, 7.0, and 8.0 to determine the optimal pH conditions. In turn, the concentration of ground *P. ostreatus* mycelium was in two variants: 2 and 20 mg/mL. Microtubes containing solutions enriched with the respective mycotoxins were incubated with the addition of ground mycelium (at the two aforementioned concentrations) for 2 h at 37 °C. It was observed that OTA degradation is associated with biotransformation; the presence of OTA derivatives after incubation (OTα) was found. Otα is a potentially non-toxic derivative of OTA, but its toxicological profile is still controversial due to the newest data suggesting genotoxic effects (Alfonso et al. 2022). Moreover, it was found that strongly acidic (12% degradation) or alkaline (50% degradation) pH negatively affected the detoxification process, and the optimum oscillated between 5 and 7 (99–98% degradation). A different situation was observed in the case of ZEN detoxification, which was probably based on the mechanism of adsorption to the fungal cell wall. No ZEN derivatives, i.e., α- and β-zearalenol, were found, so the authors suggested the possibility that the toxin was adsorbed to the cell wall of *P. ostreatus*, similar to how some toxins are adsorbed to the cell walls of yeast or bacteria (Pereyra et al. 2015; Piotrowska & Masek 2015). Additionally, the effect of pH on ZEN degradation was significant, with alkaline pH negatively affecting degradation (51% degradation), while acidic pH stimulated the process (75% degradation) (Nobre et al. 2022). Table 2 summarizes data on mycotoxin degradation using ligninolytic enzymes produced by *Pleurotus* spp.

**Table 2:** Degradation of mycotoxins by enzymes produced by *Pleurotus* spp. (in vitro)

Species	Type of enzyme	Presence of mediator	Mycotoxins	Degradation (%)	Reference
*Pleurotus eryngii*	Laccase	-	Aflatoxin B1	90	Brana et al. (2020)
	Manganese peroxidase				
*Pleurotus eryngii*	Laccase	TEMPO, PhR, SA, ABTS	Ochratoxin A	30	Loi et al. (2018)
			Zearalenone	100	
			Fumonisin B1	74	
			Deoxynivalenol	-	
*Pleurotus pulmonarius*	Laccase	ABTS, two phenolic compounds	Aflatoxin B1	81	Loi et al. (2016)
			Aflatoxin M1	100	
*Pleurotus pulmonarius*	Laccase	ABTS	Aflatoxin B1	100	Song et al. (2021)
			Zearalenone	100	
*Pleurotus ostreatus*	Manganese peroxidase	Cu, Ca, Mn cations	Aflatoxin B1	90	Yehia (2014)
Pleurotus eryngii*	-	-	Aflatoxin B1	86	Brana et al. (2017)
Pleurotus eryngii*	-	-	Aflatoxin B1	85	Haidukowski et al. (2019)
Pleurotus ostreatus*	-	-	Aflatoxin B1	94	Jackson et al. (2017)

*In vivo

**Fig. 1:**
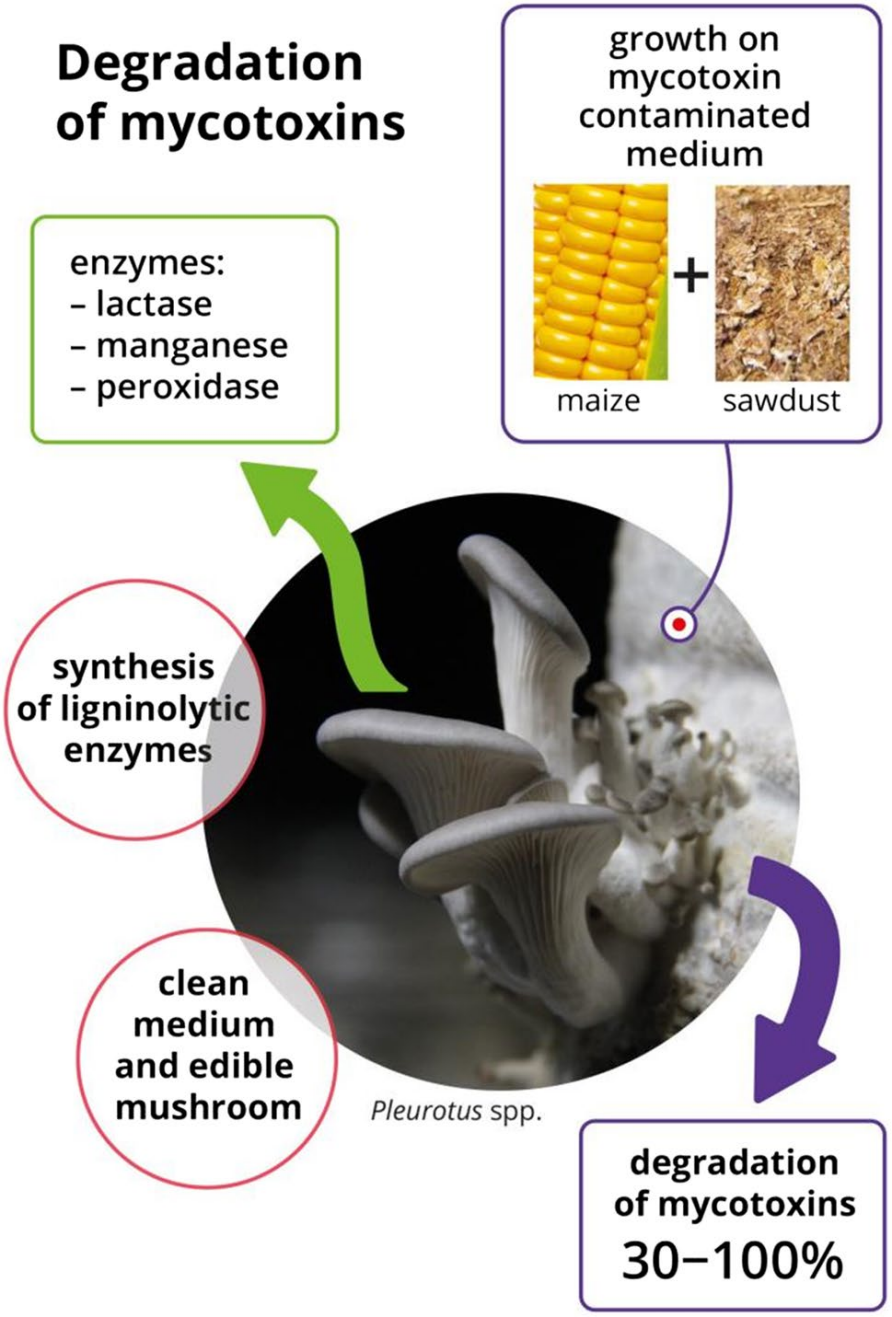
Degradation process of AFB_1_ by mycelium of *Pleurotus* spp. on contaminated medium based on the experiment proposed by Brana et al. (2017)

Among the available literature data on the degradation potential of *Pleurotus* spp. under in vivo conditions, most studies are based on reduction of AFB_1_. These studies are usually carried out using prepared substrates with the addition of straws contaminated with the selected mycotoxin. Brana et al. (2017) also considered the detoxifying effect of *P. eryngii* fungi under in vivo conditions using a ready-made culture substrate used for industrial fungal cultivation. In the cultivation carried out under laboratory conditions, fungi (*P. eryngii*) were grown using a ready-made substrate with 25% addition of AB1-contaminated corn kernels (Fig. 1). At the end of the incubation process (42 days), 86% of the toxin was degraded. In the same study, the culture substrate and mushroom mycelia were analyzed in the context of accumulation of AFB_1_ and aflatoxicol (AFOL), which is a derivative of AFB_1_ with less toxicity but can be converted back to the original substance (Detroy and Hesseltine 1970; Nakazato et al. 1990). These substances were not found in the matrices tested, excluding the possibility of toxin accumulation in the mushroom mycelia and in the substrate. Jackson et al. (2017) cultured *P. ostreatus* using a substrate with the addition of AFB_1_-contaminated corn kernels and showed no negative effect of high AFB_1_ concentration in the substrate on fungal growth. Degradation was 94%, confirming previous in vitro results regarding the high efficiency of these mushrooms to detoxify AFB_1_.

Some authors have identified other mechanisms of detoxifying activity exhibited by the *Pleurotus* spp. Research conducted by Haidukowski et al. (2019) showed an 85% reduction in AFB_1_ in the bioabsorption process using freeze-dried mycelium of *P. eryngii*. An interesting study was also conducted by Wang et al. (2022) analyzing the detoxifying potential of bicultures—a strain of filamentous fungus (*Aspergillus niger*) and *P. ostreatus* in the context of reducing AFB_1_ levels. It was shown that the enzymes produced by both species showed a strong detoxifying effect, allowing almost complete removal of aflatoxin from the substrate.

## Discussion

Taking into account the global problem of contamination with fungal secondary metabolites of raw materials, there is a need to develop new, effective biological methods for reducing these contaminants. It is important that the methods used are characterized by both high effectiveness and safety for living organisms and the environment. The presented review of research on the use of *Pleurotus* spp. shows a wide range of possibilities. So far, in laboratory studies on the reduction/degradation of mycotoxins, the *Pleurotus* spp. has been used in two different directions: (i) the degradation of mycotoxins using ligninolytic enzymes isolated from mushrooms or (ii) the cultivation of *Pleurotus* spp. on toxin-contaminated grain substrates. Both directions have high application value, but in order to fully characterize the application potential of these fungi, it is necessary to first conduct comprehensive studies of a molecular, microbiological, and metabolomic nature, which are lacking at this point. Many factors can affect the effectiveness of both processes (temperature, pH, addition of mediators and/or bioactive compounds, process duration, type of substrate, mushroom species, etc.), and their optimization is very important. Degradation of mycotoxins using enzymes from *Pleurotus* spp. requires further research, especially in the selection of mediators supporting this process and the effectiveness of the enzymes (laccase and manganese peroxidase)—each separately or in a mixture, in isolated form or not, on a liquid medium and/or solid. Literature data mainly focus on the degradation of aflatoxin B1, showing high effectiveness of ligninolytic enzymes. Such promising results regarding the degradation of aflatoxins using *Pleurotus* spp. prompt us to pay more attention on *Fusarium* toxins because the literature data in this area is very incomplete. Previous research shows that each of the *Fusarium* toxins—due to its diverse chemical structure—is degraded to a varying extent, as well as the fact that they occur in the matrix simultaneously as metabolites of different *Fusarium* species. An important aspect is also the fact that in addition to the parent forms of mycotoxins, their derivatives are also produced, often with higher toxicity. Recognizing these relationships requires the use of advanced analytical tools and should be a direction for further research in this area. In turn, the use of *Pleurotus* spp. cultivation on toxin-contaminated grain substrates may be a biological and ecological way of managing agricultural waste, including low-quality grain. An important feature of *Pleurotus* spp. is its rapid growth, the ability to grow on a variety of substrates, and the lack of selectivity in relation to the components present in the substrate. Nevertheless, it is important to evaluate the effects of different concentrations of mycotoxins on the rate of mycelial proliferation and fruiting body formation, taking into account potential changes in gene expression that may occur as a result of these substances. Moreover, taking into account previous research indicating the lack of mycotoxins in the fruiting bodies of *Pleurotus* spp., the identification of toxin degradation products and the evaluation of their toxicity should be taken into account, in which metabolomic analysis would be helpful. To fully assess the *Pleurotus* spp. potential in the process of degradation of mycotoxins, future research directions should include also diversity of *Pleurotus* spp. in terms of safety and suitability for consumption, their enzymatic composition, and optimal cultivation conditions, as well as the addition of mediators and the type of substrate.

In terms of practical applications, these methods could be integrated into food and feed processing to reduce contamination levels, contributing to enhanced food safety and lower economic losses associated with mycotoxin contamination. For instance, *Pleurotus* spp. could be used in biotechnological processes for detoxifying contaminated crops that are intended for animal consumption. The large-scale cultivation of *Pleurotus* spp. on agricultural waste materials contaminated with mycotoxins could offer an eco-friendly solution to waste management while producing edible mushrooms. However, before these methods can be widely implemented, additional research is needed to optimize cultivation conditions, assess the toxicological aspects, ensure safety, and estimate the economic feasibility of applying these techniques on an industrial scale.

## Data Availability

No datasets were generated or analysed during the current study.
